# Reconsidering ‘ethics’ and ‘quality’ in healthcare research: the case for an iterative ethical paradigm

**DOI:** 10.1186/s12910-015-0004-1

**Published:** 2015-05-08

**Authors:** Fiona A Stevenson, William Gibson, Caroline Pelletier, Vasiliki Chrysikou, Sophie Park

**Affiliations:** Research Department of Primary Care and Population Health, UCL, Rowland Hill Street, London, NW3 2PF UK; Department of Culture, Communication and Media, Institute of Education, 20 Bedford Way, London, WC1H 0AL UK; Department of Lifelong and Comparative Education, Institute of Education, 20 Bedford Way, London, WC1H 0AL UK

**Keywords:** Quality, Research ethics committees, Health research, UK

## Abstract

**Background:**

UK-based research conducted within a healthcare setting generally requires approval from the National Research Ethics Service. Research ethics committees are required to assess a vast range of proposals, differing in both their topic and methodology. We argue the methodological benchmarks with which research ethics committees are generally familiar and which form the basis of assessments of quality do not fit with the aims and objectives of many forms of qualitative inquiry and their more iterative goals of describing social processes/mechanisms and making visible the complexities of social practices. We review current debates in the literature related to ethical review and social research, and illustrate the importance of re-visiting the notion of ethics in healthcare research.

**Discussion:**

We present an analysis of two contrasting paradigms of ethics. We argue that the first of these is characteristic of the ways that NHS ethics boards currently tend to operate, and the second is an alternative paradigm, that we have labelled the ‘iterative’ paradigm, which draws explicitly on methodological issues in qualitative research to produce an alternative vision of ethics. We suggest that there is an urgent need to re-think the ways that ethical issues are conceptualised in NHS ethical procedures. In particular, we argue that embedded in the current paradigm is a restricted notion of ‘quality’, which frames how ethics are developed and worked through. Specific, pre-defined outcome measures are generally seen as the traditional marker of quality, which means that research questions that focus on processes rather than on ‘outcomes’ may be regarded as problematic. We show that the alternative ‘iterative’ paradigm offers a useful starting point for moving beyond these limited views.

**Summary:**

We conclude that a ‘one size fits all’ standardisation of ethical procedures and approach to ethical review acts against the production of knowledge about healthcare and dramatically restricts what can be known about the social practices and conditions of healthcare. Our central argument is that assessment of ethical implications is important, but that the current paradigm does not facilitate an adequate understanding of the very issues it aims to invigilate.

## Background

Most UK-based research in the healthcare context requires ethical approval from the National Research Ethics Service. The UK National Research Ethics Service and the Research Ethics Committees are mechanisms that aim to “safeguard the rights, safety, dignity and well-being of people participating in research, as well as *facilitating and promoting ethical research that is of potential benefit to participants, science and society*.” (emphasis added) [1]. This article is particularly concerned with the aim of ‘facilitating and promoting research that is ethical and beneficial’. We argue that there is considerable tension between the current definitions of ‘quality’ that form the basis for ethical review procedures, and the methodological aims of much qualitative social research. Specifically, methodological benchmarks such as ‘testing hypotheses’ and ‘measuring outcomes’ do not align with research designs that are justified on the basis of describing multiple, complex and synergistic social processes as these occur naturalistically.

Our aim in this discussion is to highlight that what constitutes a ‘quality’ application reflects disciplinary context and perspective. The ‘one size fits all’ approach to ethical reviews limits the production of knowledge about healthcare as it effectively devalues forms of enquiry that breach an implicitly, sometimes even *explicitly* preferred research paradigm. Where research is forced to modify its design in order to take account of judgements emanating from a different research paradigm it creates a potential risk to the quality of the research [2], and to the ability of researchers to maintain an ethical stance. In making this claim, we do not underestimate the complexity that more methodologically open review systems would face. However, we argue strongly that changes to ethical review are needed in order to maximise the quality of healthcare research and allow the social and cultural dimensions of healthcare provision to be informed by rigorous research evidence. Through this discussion our aim is to contribute to the body of literature that has already highlighted some of the limitations with current ethical procedures in healthcare contexts, particularly in the UK (see for instance [2-8]). A key point that is made within these debates, and which we wish to examine and re-emphasise here, is that conceptions of ‘ethical conduct’ are not methodologically neutral, but are shaped through specific disciplinary or methodological approaches.

### Ethics in the human and social sciences

The case for ethical review boards is easy enough to make. Research may be harmful to those involved in it, be that researchers, participants, or other associated parties. This necessitates mechanisms to make sure that such risks are evaluated and responded to productively. These arguments have been discussed in some detail elsewhere (See for instance [4,8,9]). In brief though, ethical review of research is regarded as justifiable because of two important historical issues; namely (a) inhumane experimentation on human subjects, particularly in Nazi Germany, and (b) research subsequently judged by some as ethically suspect, such as that which occurred in the past in the social sciences prior to ethical regulation (the most well-cited of these are Humphreys’ studies in 1970 of the sexual practices of gay men [10]; Milgram’s 1965 experiments in obedience [11], and Zimbardo’s 1974 experiments of conformity [12]). As with methodological procedures more broadly, the medical model of scientific research has been used as a standard for ethically appropriate research activity, and while there are now ethical procedures for the social sciences, the language of the natural sciences remains influential in their formulation [13]. This of course includes how ‘quality’ is both defined and applied. Further, the administration of ethical standards in research has been increasingly bureaucratized, and higher education institutions are expected to subject all research to such reviews.

In recent years, there has emerged a growing body of work that gives voice to frustrations over the difficult situation that academics find themselves in when dealing with ethical review boards. There is already plenty of evidence of the power of ethics committees to reshape research, particularly, although by no means exclusively, for qualitative research. There are many examples in the literature of research that has been prevented because of interpretations of ethical norms applied inappropriately (see for instance [7,8]). There are also a number of interrelated issues that are a source of complaint for academics undergoing ethical review:

Firstly, there is a perceived double standard within society, where academics are subject to stringent rules over how and what they can investigate, whereas institutions such as the media and the police, and indeed anyone operating outside of an institution, for example artists, can record the activities of societies members and do pretty much whatever they want with those records [4]. A similar point has been made in relation to journalism, in which the freedom of the press is used as an argument to challenge attempts to subject journalists to ethical review [6].

Secondly, the risks associated with research in the humanities and social sciences are regarded as being qualitatively different from risks in biomedical science research. Haggerty [4], Dingwall [7] and Hammersley [2] all argue that the extent of potential harm in social research is more limited than in research involving medical interventions. As Dingwall puts it “HSS (Humanities and the Social Sciences) researchers do nothing that begins to compare with injecting someone with potentially toxic green stuff that cannot be neutralised or rapidly eliminated from their body if something goes wrong. At most there is a potential for causing minor and reversible emotional distress or some measure of reputational damage” [7].

Thirdly, the ethics committees are sometimes seen as a barrier to producing responsive research, as the immense administrative burden that they place on the already restricted time that academics typically have for research limits dramatically how quickly researchers can respond to and investigate rapid social changes, particularly in points of social crisis [2,5].

Fourthly, review boards have displayed a lack of understanding and sensitivity towards the distinctive aims and interests of qualitative research and their methodological underpinnings [3,5,8]. Of particular concern is that foundational ethical principles in qualitative research, particularly research concerned with emergent research designs, grounded theory [14] or participant research [15], are impossible to implement in other forms of research, and are therefore effectively prohibited. To take one example of many possible problems, the insistence on informed consent makes ethnography largely impossible as it ‘denaturalises’ the environment being researched, turning it into a formal research encounter rather than an exploration of culture as it happens. Moreover, on a practical note, ethnographers are unlikely to have the resources to establish fully informed consent from every person they may encounter in any given setting, by virtue of the complexity of most settings. Librett and Perrone [8] suggest that because of the substantial methodological differences between qualitative work and what they describe as the ‘positivistic’ frameworks of ethics, ethnographic research should be examined under a different review process to quantitative approaches.

The concerns outlined above have resulted in a number of arguments against the presence of review boards. One common argument is that ethical review operates against the rights of autonomy for academics [7]. Tierney and Blumberg Corwin [16] have argued that there is now an inherent tension between the ideal of academic freedom and the processes of institutional review boards. They suggest that this is not just a matter of a lack of understanding of qualitative enquiry, but something much more general that relates to the very notion of academic work and identity in the new millennia. In essence, the standardisation of procedures for research through ethical guidelines diminishes the autonomy of researchers to delineate their own methods and methodological approaches or to formulate their own questions and therefore crucially to do good quality research.

Jacques Derrida has been a particularly high profile voice in defending the independence of academic research. He notes that “The University should thus also be the place in which nothing is beyond question, not even the current and determined figure of democracy, and not even the traditional idea of critique, meaning theoretical critique, and not even the authority of the “question” form, of thinking as “questioning” [17]. The reference to democracy here is important, as it is increasingly common to see social research as a component of the development and maintenance of democratic ideals, and some research guidelines (such as the British Educational Research Council) make explicit reference to this as an ethical principle. In Derrida’s view, *real* freedom, and real ethics, implies the possibility of critiquing even this.

Another common argument against ethical review committees is that they jeopardise the notion of free inquiry for society more generally, and operate against the notion of research that is not controlled by those in political positions. The UK’s ESRC is one of the main funders of social research in the country, and it has historically set the standards for ethical enquiry, both in terms of the training of researchers, but more tangibly in delineating the standards that all research that it funds must adhere to. It is not hard to see that there is some considerable tension between the notion of ‘free enquiry’ and a system where both the topics of research and the methods through which it is carried out are defined in large part by one government-funded institution [2,7].

A further argument raised by Hammersley [2] is that there is considerable lack of consensus in research communities regarding ‘appropriate ethical practice’, calling into question the idea that a collection of experienced researchers on a review panel are well placed to *resolve* ethical decisions: what is more likely, perhaps, is that researchers conflict in their opinions and compromise their views for the sake of reaching decisions. Moreover, Hammersley [2] further points to the argument that general ethical principles should be interpreted in the light of the specifics of a local research setting and methodological expertise, and generally speaking it is not the ethics committee that possesses this knowledge, but the researchers themselves. For these reasons, Hammersley suggests that ethics committees are not well placed to undertake ethical reviews: the regulatory framework within which they are embedded is counterproductive when, or because, it absolves researchers from the necessity and the practical process of reflecting on the ethical considerations raised by, and during, their research.

Consideration of ethical concerns is generally accepted as necessary and there are a range of ethical guidelines reflecting different disciplinary or methodological emphases; so for instance, the British Educational Research Association has its own ethical guidelines which differ from those, say, of the British Sociological Association or the British Psychological Association. Alongside these guidelines on what constitutes ‘ethical practice’, there are bodies charged with providing ethical reviews, such as University Ethics Committees, the Social Care Research Ethics Committee and The NHS National Research Ethics Service. Our focus here is on reviews by the latter. We argue that this system of ethical review contains an extremely rigid conception of ‘quality’, and further, that a restrictive, positivistic ethical paradigm is, in our experience, dominant within it. This is visible, for instance, in the use of the terminology of ‘human subjects’ - as singular phenomena - and the broader language of the natural and medical sciences which remains highly influential [3,5,13], and plays a particular role in delineating what is permissible within research. This terminology hides the complexity of ways in which the people who engage in research may be defined and therefore how they participate in research. Terms such as research ‘subjects’, ‘objects’, or ‘participants’ are not just alternative terms for ‘people’, but are ways of conceptualising the role of people within the research process, and have embedded within them different assumptions about the aims and purpose of the research process, and therefore what constitutes research ethics.

## Discussion

### Differing ethical paradigms

As Hammersley and Traianou [18] pointed out, there are a range of perspectives in relation to ethical review; here we outline a framework for comparing two very different ‘ideal type’ paradigms of, and approaches to, ethics in social research in relation to both the processes of knowledge production and methodology. To reiterate, our intention is not to reject the importance of ethical reviews, but to provide a critique of the current operationalisation of the NHS Research Ethics process, particularly (although, again, not exclusively) in qualitative enquiry. In undertaking this discussion, we present these two paradigms as ‘ideal types’, and show their relation to broader methodological debates in the social sciences. While we will make reference to some of these debates, it is not our intention to provide a thorough review of them– rather, our interest is in signalling the ways that they impact on considerations of ethics and, more specifically, on the role of review boards in dealing with them. Having outlined these two paradigms we consider, drawing on our recent experience of the processes involved in gaining NHS ethics, how the ideas presented map on to our experiences.

The first paradigm we label as ‘pre-dictive ethics’; the hyphenated name is intended to draw attention to the dual characteristics of ‘pre’ defining and ‘dictating’ ethical procedures. This paradigm is characterised by the following principles:

- The hypothesis to be tested is specified in advance
- The risks involved in doing the research are predictable
- The research process will either affirm or refute the starting hypotheses

The pre-dictive approach to ethics involves deciding in advance the possible research scope, remit, questions, and design and, as a consequence, regards ethical problems as something that must also be identified (and solved) *prior* to research.

In contrast to this, an ‘iterative ethics’ paradigm is characterised by the following general commitments:

- Research questions and design established at the start of the research process evolve in the light of an ongoing process of analysis
- Some risks can be specified in advance, but the researcher cannot claim to predict them all since these will emerge as a result of the research process itself, with the researcher then expected to carry out ethical reflection
- Research outcomes consist of statements made possible by the research process, rather than the acceptance or refutation of prior hypotheses

These two paradigms reflect different ways of thinking about the process of knowledge production and its relation to methodology, and imply different languages for describing this relationship. Table 1 gives an overview of the general methodological principles that are characteristic of each one. In the following discussion we will work through these issues one by one, illustrating the tensions that exist within them, and the very different methodological implications for the orientation to ethics.

**Table 1:** **Comparing two ethical paradigms**

	**Pre-dictive ethics**	**Iterative ethics**
1	Ethical risks are generally predictable	Ethical risks may NOT be predictable
2	Ethical procedures should be pre-specified - Ethics as ‘requirement’	Ethical procedures should be emergent to take into account the unfolding context - Ethics as ‘process’
3	Treats participants as being ‘subject to’ research	Treats participants as being ‘subjective participants within’ research
4	Ethics reviews aim to protect participants	Ethics reviews aim to help researchers to think sensitively about how to maintain an ethical stance towards and with research participants
5	Ethics reviews aim to evaluate researchers	Ethics reviews aim to work with researchers to explore ethical concerns
6	Ethics reviews ‘apply’ codes of conduct and treat ethics as a set of ‘accountable standards’	Ethics reviews analyse ethical concerns with researchers in relation to the specific research context
7	Researchers treated as independent from practices of data collection and regarded as implementing a research protocol	Researchers seen as reflexive participants within research
8	Work with a binary of ‘ethical’/‘non-ethical’	Treat ethical problems as multidimensional and contextually framed

1. In a pre-dictive approach, ethics is conceptualised as a set of possible eventualities that can be pre-conceived prior to the enactment of research. Research ethics are measurable and accountable, and are translated into formulations of ‘ethical behaviour’, as a pre-defined model of action. In an ‘iterative’ approach, ethics is conceptualised as an on-going practice that is realised in interaction. While some ethical concerns may and should be imagined prior to research, the iterative nature of the research means that many more ethical issues are likely to emerge, and even those that can be guessed may not be seen in the most appropriate way prior to beginning a research project. In an iterative paradigm, ethical approval is only the first step in the process of doing ethical research, ethical issues are regarded as emerging from the very process of research engagement. Just as data analysis, research design, and writing are not ‘stages’ of research but are on-going areas of work [19,20], ethics is also better thought of as a research *process* rather than a research *requirement*. This observation is not intended to suggest that ethics boards need to meet on multiple occasions; rather than a call for additional bureaucracy, the issue pertains to how ethical issues can be identified and the researcher can maintain an ethical stance in doing research. However it is managed, the current processes invoked by NHS ethics committees are not fit for purpose in ensuring ethical research where the research design is intended to enable the investigation of emergent social processes as opposed to pre-defined outcomes.
2. If researchers produce knowledge by conducting research *on* participants, it is usually treated as reasonable that the possible risks ‘for’ or ‘to’ such participants be specified comprehensively prior to their involvement. If researchers produce knowledge by conducting research *with* participants, taking into account and investigating their interpretations, questions and priorities, it is usually treated as *not* reasonable - and moreover profoundly *un*ethical - to require such researchers to predict comprehensively the risks of doing the research as well as the research outcomes. This is because such pre-specification excludes the possibility of the research process responding to emerging (and unpredictable) events in the research setting. So, for instance, in traditions such as ethnography and grounded theory, the research design is emergent and not comprehensively decided in advance, precisely to allow questions and designs to develop as the research progresses [14,15].
3. The two ‘ideal types’ of research traditions have implications for how ethical committees consider participants in research. In pre-dictive approaches, attention is given to research participants as ‘objects’ of the research process, and, consequently they tend to be viewed as potential ‘victims’ of any unethical practices perpetrated by the researcher. The role of the committee is, then, to protect participants and assume the role of the ethical arbiter, protector and judge. By contrast, in iterative traditions, attention is given to research participants as subjects *in* the research process (in the sense of ‘subjective participants in’ rather than ‘subject to’) and are treated as helping to shape the research process, including the formulation of research questions and the data collection process. The reflexive turn in the social sciences; the emphasis on the mutual construction of discourse; the positioning and negotiation of subject identities and the instability of knowledge as a category –have all profoundly influenced debates about the nature of relations between researchers and research participants. While there are many manifestations of this debate, one of the most famous overtly methodological articulations is Charmaz’ [21] outline of ‘constructivist’ grounded theory. The general point we wish to emphasise is that qualitative methodologies have moved a long way from the visions of research participants as passive recipients of research actions, to a view of knowledge (and therefore ethics) as a joint problem, not a researcher (or ethics committee) problem. To pre-empt a potential complaint, this issue is not solved by having representation from user groups on a review panel (e.g. patients): what is required is a different way of thinking about how ethical issues emerge through research.
4. As a consequence of point three, in pre-dictive approaches to ethics the intention is to analyse the possible risks that a research participant may encounter, and to make sure that the researcher has a sufficiently developed strategy for dealing with them. In iterative ethics, the review board’s role involves taking into account the interests of research participants but does not imply specifying these interests comprehensively in advance *for* them. It thus acknowledges participants’ autonomy as decision-makers and their capacity to make judgements based on their own ethical stance within the research setting.
5. The predictive nature of ethical problems means that the role of ethics boards in the first tradition is to evaluate the extent to which researchers have been able to effectively foresee ethical problems, and to design an ethically appropriate research protocol. In iterative approaches, because ethics are not ‘pre-conceived’ their role is facilitative, aiming to help researchers to think through possible problems, and to act as something like a ‘critical reader’ rather than a judge.
6. The shift implied in point five means that there is a stark difference between, on the one hand, viewing ethics as a set of procedures to be applied to researchers, and on the other, treating ethics as a matter to be discussed and analysed in relation to the context. This shift in perspective to ‘working with’ rather than ‘evaluating’ researchers is a crucial and positive part of the role that research committees could, but currently do not generally take. To phrase the matter like this may sound like the committees should lose the capacity to block research or to make changes to it; this is not what we are suggesting. Rather, the idea is that the basis of the judgement on which such blocking may occur needs to change from ‘the researcher’s ability to describe, predict and avoid hypothetical ethical problems’ to ‘the researcher’s ability to reflect on the ethical sensitivities of a setting, and to devise strategies for managing them’. Thus the emphasis should be on the quality of ethical thinking, rather than the ability to pre-specify a long list of potential scenarios with research applications demonstrating critical reflexivity, not a list of commitments which are then treated as exhaustive. This shift in the role of a panel has important implications for their possible formation. If such boards are to act as anything other than the implementers of pre-conceived ethical protocols, then their structure would need to be representative of the methodological communities that they are representing. In summary, we argue for a conception of ethics as an ongoing, integral part of the research, as opposed to a one-time assessment resulting in research being judged as ‘ethical’ or not. We are not arguing for the extension of ethical regulation, but rather for recognition by ethical committees of the emergent nature of ethical risks and the creation of a cooperative environment in which this can be considered and discussed.
7. These positions carry through into considerations about the position of the researcher in relation to the research. In the first tradition, the researcher is understood to be largely independent from practices of data collection; they are treated as implementing a research protocol, rather than interacting with the object of research. Although there is increasing interest in relation to the possible effect of the researcher on data collected, this generally leads to attempts to minimise rather than accept and embrace such effects as intrinsic. In the second tradition, the researcher is understood to be an instrument within the research, a reflexive participant involved in the mutual production of knowledge, making sense of a research setting in interaction with research subjects. The presence of the researcher is not incidental to, but rather definitive of, not only what data are collected and how these are interpreted, but also of the ethical issues that are raised.
8. Finally, in a pre-dictive paradigm, research is subject to a binary of ‘ethical’ versus ‘unethical’, and the aim of the board is to ensure that research that does not meet its protocols is not allowed to proceed with a judgement that it is ‘unethical’. However, for iterative research, ethics are not seen in this way, but rather are treated as relational and multidimensional. The research process as a whole is not considered straightforwardly either ‘ethical’ or ‘unethical’. Instead, attention is placed on how the researcher defines, negotiates and maintains an ethical stance, and how they manage (often with participants) ethical considerations in a research context characterised by the often multiple ethical frameworks of participants, as well as research ‘users’. As a consequence, the ethics of a research project are not totally controllable by the ethics committee, but much more dependent on the researcher and multiple others, including research participants. Therefore, time spent engaging with an ethical committee is but one of many (and possibly more significant) ways of ensuring that research is ethical.

### The implications for judgements of ‘quality’ in research

We now move to ground the ideas presented by reflecting on some key incidents from a recent experience of seeking ethical review for a piece of ethnographic research, best fitted to ‘iterative ethics’. We focus in particular on the ways in which responses from the committee were couched in terms of quality.

Our study’s initial aim was to investigate empirically how ‘decision-making’ was done in practice in an A & E setting. This interest arose from two main concerns: first, the limited amount of observational, empirical research on decision-making, and shared decision-making specifically, in healthcare which can serve as a basis for teaching decision-making in medical education (the research team all have some connection to clinical education); second, the view expressed to us by A&E consultants that foundation year doctors had difficulties making decisions. On the basis of these concerns, we designed a study which would allow us to examine two things: the activities and practices constituting ‘decision-making’; and the involvement and participation of junior doctors in such activities and practices. The first part was intended to be an ethnographic consideration of decision making in an A & E setting, and the second part focused more specifically on the actions of particular participants – junior doctors – with observations of a small number of consultations led by them, supplemented with interviews. The data we proposed to collect comprised observational field notes, video recordings of doctor patient consultations, as well as video and audio recordings of doctors’ working routines. Such data have previously been collected in related studies carried out in other countries [22-24]. Our study was designed as a pilot to research how ‘decision-making’ might be investigated empirically in A&E and other kinds of healthcare settings.

#### Judgements about research design

Underpinning the objections raised by the research committee to aspects of our research design lay their concerns about the overall value and quality of the research. Specifically, the committee judged that an interest in decision-making necessitated our making judgements about good and bad decisions, and that our failure to measure this was a sign of poor quality research. Our emphasis on researching *processes* of decision-making, rather than the quality of decisions, caused particular problems when seeking a favourable ethical opinion. The committee decided that balanced against the intrusive nature of the data gathering and the ethical risks to patients of having their actions and cases recorded, this topic was of questionable value - as we were not proposing to monitor ‘safe practice’ within the setting in return for participation. Associated with this was the concern that the project should monitor ‘good practice’ within the medical settings. A member of our team is medically trained, and the ethics committee saw her as having an important role in ensuring that all practice observed as part of the research was ‘good practice’*.* This is significant, as in making this recommendation, the committee effectively privileged *judgement* of medical practice (in relation to biomedical, procedural norms) over a consideration of the processes involved in the construction of medical practice. In other words, the concern to make sure that the doctors were adhering to protocols was seen as more important than an investigation of how those protocols were translated in practice. The concern to protect participants (points 3 and 4), by insisting on ensuring that medical protocols were adhered to, meant that we were asked to transform the nature of the study from one looking at how medicine is practised, to one focussing on measuring how well it is practised by junior doctors. Our methodological and ethical commitments as researchers, as well as the concerns of the clinicians in the setting with which we had collaborated in developing the initial design, were overruled. Moreover, there is another, broader ethical issue relating to the procedure proposed by the review board. This it to what extent can a researcher, even one that is medically trained, judge a (nother) clinician’s practice on the basis of limited data, particularly given the wider context within which all care takes place?

#### Implications of judgements about research design

The feedback and discussions which emerged during the process of ethical review indicated that the ethics committee found the proposed ethnographic and iterative research design to investigate our questions of the process of decision making problematic. This was expressed in several ways. In relation to point 1 in our table, we were told to specify precisely the inclusion and exclusion criteria for categories of patients to be involve in our research; our broad criteria, pertaining to the ability to consent to participation as well as our own judgement and that of a senior clinician about who to approach for consent, was rejected. This may seem trivial, but required us to construct a list of people defined by medical criteria that would be excluded, despite the fact that in practice people judged unable to give consent for video recording will be excluded, regardless of their stated medical problems. Crucially, what was effectively dismissed in this request to change our selection criteria was our ability, as well as that of our clinical colleagues and patients themselves, to make judgements in the setting about the ethical risks raised by participation; the committee demanded that we predict the risks so that they might then pass judgement on them (see points 5, 6 and 7 in our table). This meant that a wide range of patient categories were then excluded from our research protocol, preventing, for instance, any possibility of identifying iteratively categories of patients for which processes of ‘decision-making’ might raise distinctive issues. It also meant that there was little possibility to discuss ethical concerns about possible but unpredictable situations, since the committee treated risks which were not predicted as a sign of poor research.

We were also asked to seek individual written consent from all participants for each instance of observation, on the basis that participants needed to be fully informed about the risks of participation prior to consenting to it. This request is problematic for ethnographic research, in which researchers participate in and observe settings naturalistically, and where consent procedures are organised to support this (for instance, seeking consent verbally, and in ways which are appropriate to the unfolding context – see point 2 in our table). This request has required us to reduce significantly the scope of our initial phase of observation, and focus most of our time and attention on the activities of a pre-specified group, namely junior doctors. There are two potential dangers with this amendment to our research design. The first is that it significantly restricts our ability to investigate decision-making as a complex, social process involving multiple actors across the A & E setting, as suggested by Iedema et al.’s [22] work carried out in Australia and Berg’s [25] study in the Netherlands, which point to the socially distributed and temporally extended nature of decision-making. The second danger is that it leads us to focus on the activities of one group – junior doctors – with limited ability to contextualise these within wider social processes; the risk is that this group is pathologised in relation to medical decision making by virtue of being the main focus of the research. Thus junior doctors’ actions becomes the focus as opposed to understanding their decision making as shaped and to some extent determined by the complexity of the wider context within which it occurs.

#### Who decides what is ethical?

The institutional role of the ethics committee as gatekeeper to research with the right to decide what is ethical or not, manifested very problematically for us in one particular episode. We were advised that if a participant withdrew from the study, their data could be withdrawn too, as we had proposed. However, we were told that we would need to retain the data and make it available in cases of a relevant legal complaint or procedure. This recommendation raises for us a profound ethical issue relating to the confidentiality of participant data and the nature of the researchers’ promises to participants (including patients) regarding its security. It meant that we could make very few promises about the confidentiality and safeguarding of data (e.g. limiting who can see it, where it will be used, how it will be stored): the data were treated as available to the hospital upon its request and under conditions which are not subject to ethical review. On the one hand then, we were asked to offer participants confidentiality and anonymity, and on the other, the possibility for respecting such offers was withdrawn. Although this seemed a paradox to us, it was not perceived as such by the committee, a perception dependent upon treating only research and researchers (versus other authorities including hospital management) as presenting ethical risks in their interactions with staff, patients and also researchers. The judgement of these other parties, about ethical risks and where these might lie was not treated as relevant.

In the assessment of our proposal the wider implications of enforcing changes to our research design that narrowed its focus was not recognised by the ethics committee as their concern was directed at the risks presented by participation in research activities, and not by the wider implications on what could then be known. In other words, the risks to junior doctors were treated as pertaining to the conditions under which consent would be requested, and not to the formulation of the problem which the research was intended to address. This reflects the ethical/non-ethical binary within which the committee was working, and which excluded the possibility of research presenting ethical problems at different levels, multidimensionally, and according to context (point 8 in our table).

To reiterate, the main point being made here is not so much in relation to the decisions themselves, but the basis on which ethical research practice was determined. The ethical risks presented by the study were treated as subject exclusively to the decisions and definitions of the ethics committee. The commitments and concerns of the researchers as well as those in the setting (including patients as potential participants) were not treated as relevant. For instance, the possibility of patients deciding on the value of the research in context and iteratively with researchers was pre-empted.

After several iterations of the review process, our application was finally approved. In the process, our research questions and design were re-shaped to reflect the predictive ethical paradigm within which the committee operated. In some respects, this has forced us to clarify aspects of our design, but in many others, it has narrowed the scope of our research aims, and set up several ethically problematic conditions for doing the research (problematic for us). It has also consumed the entire initial research budget for doing the work. Insofar as this is a pilot study, such results are themselves interesting, but they raise many questions for us: what scope is there to do healthcare research in the UK focusing on the quality of existing practice which is iterative and emergent in its design and its ethics? What responsibility do NHS ethics committees have in considering the risks they create for researchers through the general focus on a single ethical paradigm, which privileges questions concerned with outcomes rather than process at a time when questions have been raised about culture and its effect on quality in health care [26]? Who is protected in ethics committees’ judgements and for what reasons? How democratic or accountable are NHS ethics committees in making judgements about the quality of research they approve? What are the effects of the NHS ethics review process on the quality and range of healthcare research and what are the implications for research-informed practice? These questions are important as the notion of quality held by ethics committees dictates what research can be done, while even projects which are approved are potentially left with the indelible mark of judgements made in the course of approval.

## Summary

In this paper we have built on existing debates and related this to recent experiences seeking ethical approval for an ethnographic study. We conclude that although there is a strong argument for the existence of institutionalised ethical regulation, a ‘one size fits all’ standardisation of ethical procedures and approach to ethical review - based on what is perceived to be good quality research with familiar, measurable outcomes - acts against the production of knowledge about healthcare, and dramatically restricts what can be known about the social practices and conditions of healthcare, including its quality and complexity. Our central argument is that assessment of ethical implications is important, but that the current paradigm and crucially its current operationalisation do not facilitate an adequate understanding of the very issues it aims to invigilate.

## References

[1] Health Research Authority. Summary of the role, structure and functionality of Research Ethics Committees within the Health Research Authority in England. 2013. [http://www.hra.nhs.uk/documents/2013/10/national-research-ethics-service-summary-ver-1.pdf]

[2] Hammersley M (2009). Against the ethicists: on the evils of ethical regulation. Int J Soc Res Method.

[3] Van den Hoonaard WC (2003). Is anonymity an artifact in ethnographic research?. J Acad Ethics.

[4] Haggerty KD (2004). Ethics creep: governing social science research in the name of ethics. Qual Sociol.

[5] Hemmings A (2006). Great ethical divides : bridging the gap between institutional review boards and researchers. Educ Res.

[6] Dash L (2007). Journalism and institutional review boards. Qual Inq.

[7] Dingwall R (2008). The ethical case against ethical regulation in humanities and social science research. Twenty-First Century Society.

[8] Librett M, Perrone D (2010). Apples and oranges: ethnography and the IRB. Qual Res.

[9] Israel M, Hey I (2006). Research ethics for social scientists.

[10] Humphreys L (1970). Tearoom trade. Society.

[11] Milgram S (1965). Some conditions of obedience and disobedience to authority. Hum Relat.

[12] Zimbardo P (1974). On “obedience to authority”. Am Psychol.

[13] Perry KH (2011). Ethics, vulnerability, and speakers of other languages: how university IRBs (do not) speak to research involving refugee participants. Qual Inq.

[14] Ramcharan P, Cutcliffe JR (2001). Judging the ethics of qualitative research: considering the “ethics as process” model. Health Soc Care Comm.

[15] Pritchard IA (2002). Travelers and trolls. Practitioner research and institutional review boards.. Educ Res.

[16] Tierney WG, Blumberg Corwin Z (2007). The tensions between academic freedom and institutional review boards. Qual Inq.

[17] Derrida J, Trifonas P, Peters M, Peters M (2005). The future of the profession or the unconditional university (thanks to the “humanities,” what could take place tomorrow). Deconstructing Derrida: tasks for the new humanities.

[18] Hammersley M, Traianou A. An alternative ethics? Justice and care as guiding principles for qualitative research. Sociological Research Online. 19 (3): 24. <http://www.socresonline.org.uk/19/3/24.html> doi: 10.5153/sro.3466.

[19] Gibson WJ, Brown A (2009). Working with qualitative data.

[20] Glaser B, Strauss A (1967). The discovery of grounded theory: strategies for qualitative research.

[21] Charmaz K, Denzin NK, Lincoln YS (2000). Grounded theory: objectivist and constructivist methods. Handbook of qualitative research.

[22] Iedema R, Mesman J, Carroll K (2013). Visualising health care practice improvement: innovation from within.

[23] Carroll K, Iedema R, Kerridge R (2008). Reshaping ICU ward round practices using video-reflexive ethnography. Qual Health Res.

[24] Iedema R, Long D, Forsyth R, Lee BB (2006). Visibilising clinical work: video ethnography in the contemporary hospital. Health Sociol Rev.

[25] Berg M (1997). Rationalizing medical work: decision-support techniques and medical practices.

[26] Report of the mid Staffordshire NHS foundation trust public inquiry 2013. [http://www.midstaffspublicinquiry.com/report]

